# Mirror imaging of impacted and supernumerary teeth in dizygotic twins: A case report

**DOI:** 10.4317/jced.51815

**Published:** 2015-02-01

**Authors:** Michele Cassetta, Federica Altieri, Alessandra Giordano

**Affiliations:** 1 Assistant Professor, Department of Oral and Maxillofacial Sciences, “Sapienza” University of Rome , Italy , School of Dentistry; 2 Research Assistant, Department of Oral and Maxillofacial Sciences, “Sapienza” University of Rome , Italy , School of Dentistry

## Abstract

Background: Mesiodens is the most common type of supernumerary tooth found in the premaxilla. It might be discovered by the clinical examination by chance on a radiograph or as the cause of an unerupted maxillary central incisor. The genetic transmission of supernumerary and impacted teeth is poorly understood. Mirror imaging in twins has been reported frequently in relation to several unilateral dental anomalies including mesiodens. This phenomenon is the appearance of an asymmetrical feature or anomaly occurring on the right side of one twin but on the left side of the other twin. The event of mesiodens mirror imaging in monozygotic twins has been described in literature. Results: This is the first reported case of mesiodens mirror images in dizygotic twins. The de-scribed mesiodens caused the eruption failure of maxillary permanent incisors. The super-numerary teeth were removed to facilitate the spontaneous eruption of the impacted perma-nent maxillary incisors. Clinical Implications: Studies related to supernumerary teeth can be useful to clinicians in the early diagnosis of this anomaly. Clinical and radiographic examinations provide a correct therapeutic approach.

** Key words:**Supernumerary teeth, twins, dental development.

## Introduction

A supernumerary tooth is a developmental numerical anomaly consisting in the presence of a tooth in addition to the normal series (1) .The mesiodens is the most frequent of the supernumerary teeth and is located in the maxillary central incisor region (2-4). An estimated 0.15-1.9 % of the population shows the occurrence of this anomaly (1-3).

Mesiodens could be discovered accidentally during a dental radiological examination.

The diagnosis usually occurs between 7 and 9 years of age. This is probably due to permanent central incisors eruption at this stage: the complaint of non-eruption induces a radiological examination that might reveal the presence of a mesiodens (5). This dental anomaly has been reported to cause delay or failure of eruption of the permanent incisors in 28% to 52% of reported cases (6,7).

The familial pattern of mesiodens in twins strongly supports a genetic influence, possibly resulting from an autosomal dominant inheritance (1,2).

Mirror imaging in twins has been reported frequently in relation to several unilateral dental anomalies including mesiodens (1-4). This phenomenon has been found only in monozygotic twins.

The aim of this study is to report the occurrence of mesiodens with mirror images in dizygotic twins, which has never been described in literature before.

## Case Report

Two dizygotic twins (a male and a female) were referred to the Department of Orthodontics of “Sapienza” University of Rome when they were 9 years old; their chief complaints were related to missing maxillary central incisors. Their medical and dental histories showed no systemic diseases and no facial trauma.

**Figure 1:**
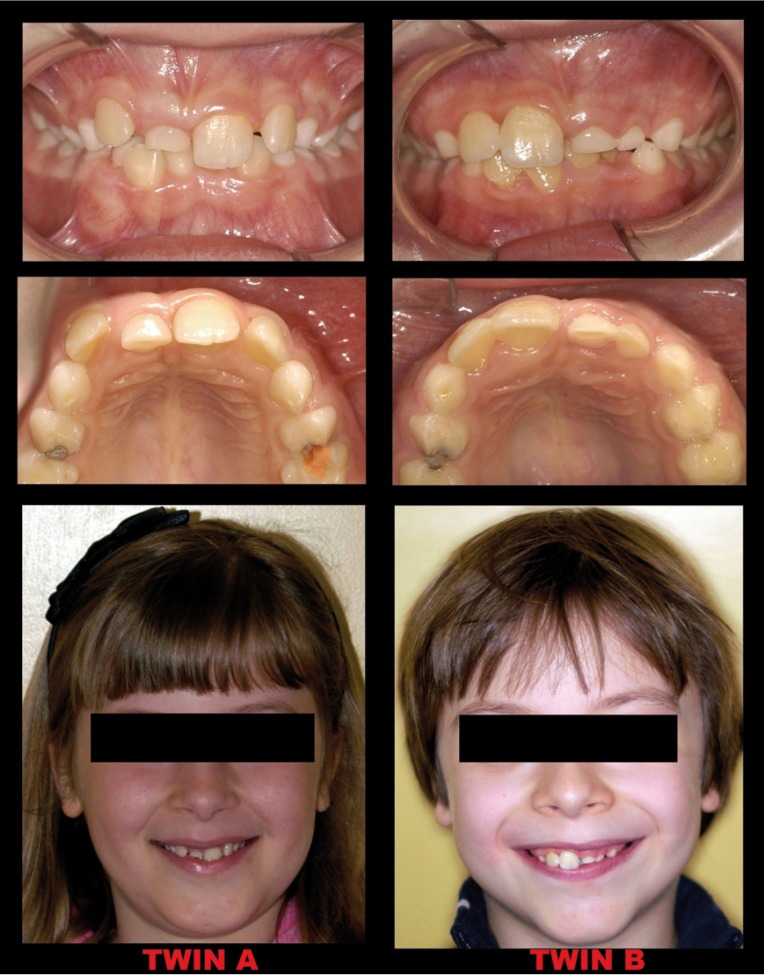
Intra-oral (frontal and occlusal) and extra-oral views.

CR (twin A, female) was in mixed dentition with the maxillary right central incisor missing. The maxillary right deciduous incisor was in the arch (Fig. 1).

AR ( twin B, male) was in mixed dentition, with maxillary left central and lateral incisors missing and the maxillary left deciduous incisors (central and lateral) in the arch (Fig. 1).

**Figure 2:**
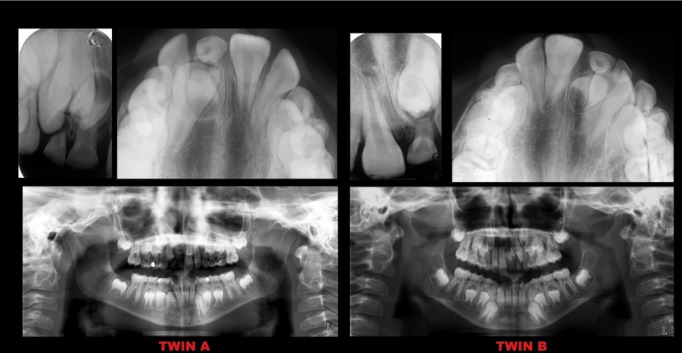
Panoramic x-ray, periapical and occlusal radiographs.

Both twins were subjected to radiographic examinations (panoramic x ray) for the investigation of unerupted teeth (Fig. 2).

The radiograph found that twin A had an impacted upper right central incisor and a mesiodens that prevented its eruption; twin B showed essentially a mesiodens mirror image with impacted maxillary left central and lateral incisors.

Occlusal and periapical radiographs were performed to evaluate size, shape and side of supernumerary teeth (Fig. 2).

These exams revealed that the mesiodens were palatal to the impacted incisors (Fig. 2).

After discussing possible therapeutic options with the parents, it was decided to perform the surgical extraction of mesiodens waiting for spontaneous eruption of the impacted permanent teeth .

The surgical technique was performed under local anesthesia. Initially , after a sulcular incision , the overretained deciduous teeth were extracted and a mucoperiosteal flap was raised to the minimum necessary extent.

**Figure 3:**
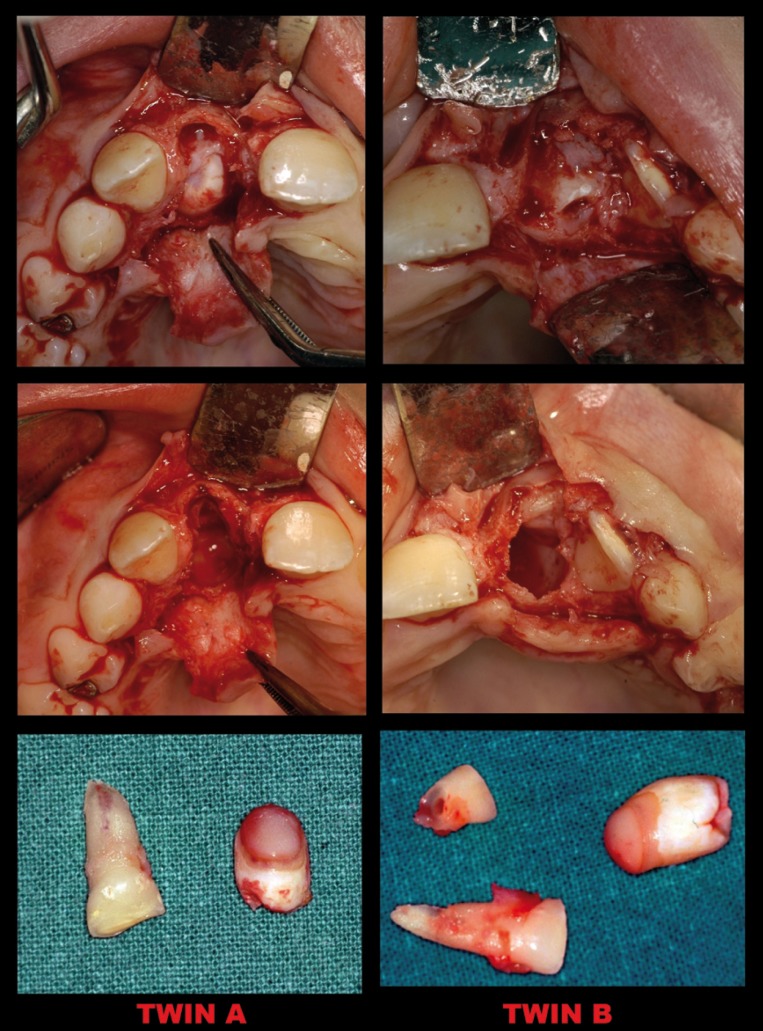
Surgical steps.

The mucoperiosteal soft tissues underlying the permanent central incisors were removed. When necessary, the bone which covered the dental crowns was removed with surgical round burs to expose the labial surface. The supernumerary teeth were extracted, and, after cleaning the area and achieving hemostasis, the flap was repositioned and sutured (Fig. 3).

After 1 week, the sutures were removed .

## Discussion

The genetic transmission of supernumerary and impacted teeth is poorly understood.

The etiology of mesiodens is still (3) not clearly established in the literature (1-6). The pathogenesis of mesiodens has been attributed to various theories such as hyperactivity of the dental lamina, a phylogenetic relic of extinct ancestors, a dichotomy of tooth buds heredity, and some environmental factors (1,2,6,8-10). The familial pattern of occurrence of mesiodens in twins strongly supports a genetic influence; many authors have focused on the genetic influence, reporting a much higher frequency of supernumerary teeth among first-degree relatives than in the general population, suggesting a significant genetic component in the etiology (1-4).

Although no investigation proved the hereditary condition of mesiodens, genetics is also thought to contribute to its development, considering that the occurrence has been diagnosed in twins, siblings, and sequential generations of a single family (8,10,11).

However some differences observed in the twins dentition suggested that environmental factors may also affect the formation of the phenotype (1-5).

The most common supernumerary teeth – those in the anterior midline – can also cause the most severe problems (e.g. delayed eruption or impaction of the central incisors). The maxillary central incisors are the third most commonly impacted teeth in Caucasians, following the third molars and the maxillary canines (9). The impaction of a central incisor is often related to an untreated mesiodens, as in this case. The treatment of choice most often involves extraction of the mesiodens and spontaneous or forced eruption of the maxillary central incisor. Thus, early diagnosis may simplify treatment and improve prognosis.

In this study the twins showed concordance considering the occurrence of hyperodontia, but they differed in the number of impacted teeth.

Twin B showed essentially a mirror image of his sister but also the impaction of maxillary left lateral incisor.

This finding can arise from the larger mesiodens size which reflects the sex differences in craniofacial morphology (12) .

An interesting aspect found in monozygotic twins is the occurrence of mirror imaging. This phenomenon is the appearance of an asymmetrical feature or anomaly occurring on the right side of one twin but on the left side of the other twin. Mirror imaging in monozygotic twins has been reported frequently in relation to several unilateral dental anomalies including mesiodens (1-6). This aspect of concordance in dizygotic twins has never been found. A review of the literature (2) reporting the occurrence of mesiodens in monozygotic and dizygotic twins showed that mirror images had been found solely in monozygotic twins. This might be due to chance or more probably to a genetic influence.

In conclusion, the occurrence of mesiodens mirror imaging in dizygotic twins, never described before, was reported.

The early detection and a correct therapeutic approach allow to solve this dental anomaly and to promote proper dental eruption.

## References

[1] Babacan H, Öztürk F, Polat HB (2010). Identical unerupted maxillary incisors in monozygotic twins. Am J Orthod Dentofacial Orthop.

[2] Seddon RP, Johnstone SC, Smith PB (1997). Mesiodentes in twins: a case report and a review of the literature. Int J Paediatr Dent.

[3] Cassetta M, Altieri F, Giansanti M, Di-Giorgio R, Calasso S (2014). Morphological and topographical characteristics of posterior supernumerary molar teeth: an epidemiological study on 25,186 subjects. Med Oral Patol Oral Cir Bucal.

[4] Langowska-Adamczyk H, Karmańska B (2001). Similar locations of impacted and supernumerary teeth in monozygotic twins: a report of 2 cases. Am J Orthod Dentofacial Orthop.

[5] Mukhopadhyay S (2011). Mesiodens: a clinical and radiographic study in children. J Indian Soc Pedod Prev Dent.

[6] Hattab FN, Yassin OM, Rawashdeh MA (1994). Supernumerary teeth: report of three cases and review of the literature. ASDC J Dent Child.

[7] Nik-Hussein NN (1990). Supernumerary teeth in the premaxillary region: its effects on the eruption and occlusion of the permanent incisors. Aust Orthod J.

[8] Townsend GC, Richards L, Hughes T, Pinkerton S, Schwerdt W (2005). Epigenetic influences may explain dental differences in monozygotic twin pairs. Aust Dent J.

[9] Brand A, Akhavan M, Tong H, Kook YA, Zernik JH (2000). Orthodontic, genetic and periodontal considerations in the treatment of impacted maxillary central incisors: A study of twins. Am J Orthod Dentofacial Orthop.

[10] Vecchione Gurgel C, Soares Cota AL, Yuriko Kobayashi T, Moura Bonifácio Silva S, Aparecida Andrade Moreira Machado M, Rios D (2013). Bilateral Mesiodens in Monozygotic Twins: 3D Diagnostic and Management. Case Rep Dent.

[11] Gallas MM, García A (2000). Retention of permanent incisors by mesiodens: a family affair. Br Dent J.

[12] Perillo L, Isola G, Esercizio D, Iovane M, Triolo G, Matarese G (2013). Differences in craniofacial characteristics in Southern Italian children from Naples: a retrospective study by cephalometric analysis. Eur J Paediatr Dent.

